# Simultaneous Detection of P300 and Steady-State Visually Evoked Potentials for Hybrid Brain-Computer Interface

**DOI:** 10.1371/journal.pone.0121481

**Published:** 2015-03-27

**Authors:** Adrien Combaz, Marc M. Van Hulle

**Affiliations:** Laboratoriumvoor Neuro- en Psychofysiologie, KU Leuven, Leuven, Belgium; Georgia State University, UNITED STATES

## Abstract

**Objective:**

We study the feasibility of a hybrid Brain-Computer Interface (BCI) combining simultaneous visual oddball and Steady-State Visually Evoked Potential (SSVEP) paradigms, where both types of stimuli are superimposed on a computer screen. Potentially, such a combination could result in a system being able to operate faster than a purely P300-based BCI and encode more targets than a purely SSVEP-based BCI.

**Approach:**

We analyse the interactions between the brain responses of the two paradigms, and assess the possibility to detect simultaneously the brain activity evoked by both paradigms, in a series of 3 experiments where EEG data are analysed offline.

**Main Results:**

Despite differences in the shape of the P300 response between pure oddball and hybrid condition, we observe that the classification accuracy of this P300 response is not affected by the SSVEP stimulation. We do not observe either any effect of the oddball stimulation on the power of the SSVEP response in the frequency of stimulation. Finally results from the last experiment show the possibility of detecting both types of brain responses simultaneously and suggest not only the feasibility of such hybrid BCI but also a gain over pure oddball- and pure SSVEP-based BCIs in terms of communication rate.

## Introduction

Brain-Computer Interfaces (BCIs) decode the brain activity with the aim to provide a direct communication channel with an external device. In this study, the brain activity is recorded using electroencephalography (EEG), which offers the advantage over other methods (*e.g*. micro electrodes, fMRI …) of being non-invasive and easy to set up.

Some of the earliest EEG-BCI systems were based on the P300 component of the event-related potential (ERP, [1, 2]). The P300 is a positive deflection in the EEG signal, time-locked to salient stimuli presented in an *oddball stimulation paradigm*. Typically, it is evoked over the parietal cortex, and occurs between 200 and 500ms after stimulus onset [3]. Such system have been shown to work successfully on both healthy and disabled subjects [4–6] and to be able to encode a large amount of targets (up to 72 targets in [7]). However, they can be slow as they rely on multiple repetitions of a stimulation sequence in order to increase the signal-to-noise ratio of the ERP; and the communication speed decreases when the number of stimuli (*i.e*. number of choices available to the user) increases.

Other systems of interest are BCIs based on steady-state visually evoked potentials (SSVEPs). They rely on the psychophysiological properties of the EEG responses recorded from the occipital cortex during the periodic presentation of identical visual stimuli (*ie*. flickering stimuli). When the latter is at a sufficiently high rate (>6Hz), stable and synchronized neural oscillations at the stimulus frequency and its harmonics are evoked over the visual cortex [8–10]. Several SSVEP-based BCIs have been successfully tested on healthy subjects and locked-in patients (see *e.g*.[6, 11], and [12] for a review). Such BCIs have the advantage to provide a relatively fast detection, however, particularly when working with on-screen stimulation, the number of usable stimulation frequencies and thus the number of choices available to the user is limited [13].

Recently, the BCI community started to develop *hybrid BCIs*, which combine different BCI paradigms in order to improve the user experience of the system. As defined in [14], *“a typical hybrid BCI is composed of one BCI and another system (which might be another BCI), and must also achieve specific goals better than a conventional system”*. The improvement achieved by a hybrid system can result in a higher accuracy, a larger number of choices, a higher selection speed, the access to a no-control state, a better usability and/or a higher number of person effectively able to use the system (BCI illiteracy, [15]). In this study, we are interested in the case where both systems are EEG-BCIs based on respectively P300 and SSVEP detection.

A hybrid system can be designed in such a way that both BCI paradigms encode the same command. Although this type of design can appear redundant, such a system could help decreasing BCI illiteracy (subjects could compensate a weak control with one modality by a better control with the other) and/or increase the detection accuracy [15]. For example, [16] combines the detection of a P300 response with the detection of an interrupted SSVEP response in a nine targets BCI to improve the accuracy of the BCI.

In another example of hybrid systems, one BCI encodes all commands available to the user, while the other BCI encodes only a subset of those commands, providing in this way additional information to help the system to identify correctly the user’s selection. This is the case of [17] where the authors use SSVEP activity to increase the performance of an P300-based spelling BCI.

A last example of a hybrid design is where each BCI provides the system with independent commands. For example, [18] proposed a system combining P300-based spelling with a single frequency SSVEP-based control-state detection where commands detected by the P300 speller where validated only if the SSVEP system detected that the subject was in a control state.

In this work, we study the feasibility of a novel hybrid P300-SSVEP paradigm for communication with multiple SSVEP frequencies where each paradigm encodes a specific and independent part of the command selected by the user so that each command can be uniquely identified by a single combination of the outcome of both detection system. Potentially, the described combination could result in a system being able to operate faster than a purely P300-based BCI and encode more targets than with a purely SSVEP-based BCI. We study the interactions between the brain responses of the two paradigms, and explore the possibilities of such a hybrid BCI, in a series of experiments where EEG data are analysed offline. This is a necessary first step before studying differences in online performances between a hybrid system and purely P300- and SSVEP- based ones (recently published work [19] demonstrates the online feasibility of such hybrid BCI and its superiority over traditional P300- and SSVEP-based BCIs).

We report in this study on three series of experiments conducted in order to 1) study the effect of a SSVEP stimulation at different frequencies on the P300 response and its detection accuracy, 2) study the effect of an oddball stimulation on the SSVEP response at different frequencies, and 3) assess the possibility to detect simultaneously the brain activity evoked by both paradigms in a proof-of-concept experiment.

## Materials and Methods

### Material

The EEG signals were recorded using a BioSemi Active Two system with 32 electrodes (following the 10-20 international system) at a sampling rate of 1024Hz. Two additional electrodes were positioned on the right and left mastoids and the mean of the signals recorded at those two sites was used to reference the activity measured by the 32 electrodes. The study was reviewed and approved by the “Commissie Medische Ethiek van de Universitaire Ziekenhuizen KULeuven” (Medical Ethics Committee, S52112). All participants provided written informed consent prior to their participation in the study.

All stimuli were visually presented on a laptop’s LCD screen (60Hz refresh rate) using MATLAB and the *Psychophysics Toolbox Extensions*[20, 21] for precise display and timing. The EEG data were processed using MATLAB and all statistical analyses were performed using R [22] and mixed effect models [23] were fitted with the R package *lme4*[24].

### Experimental protocol

#### Experiment 1: Pure oddball *vs*. hybrid ERPs

The aim of the first experiment was to study the effect of a flickering background on the typical P300 response. Nine subjects participated in the experiment (S1–S9).

**Fig 1:**
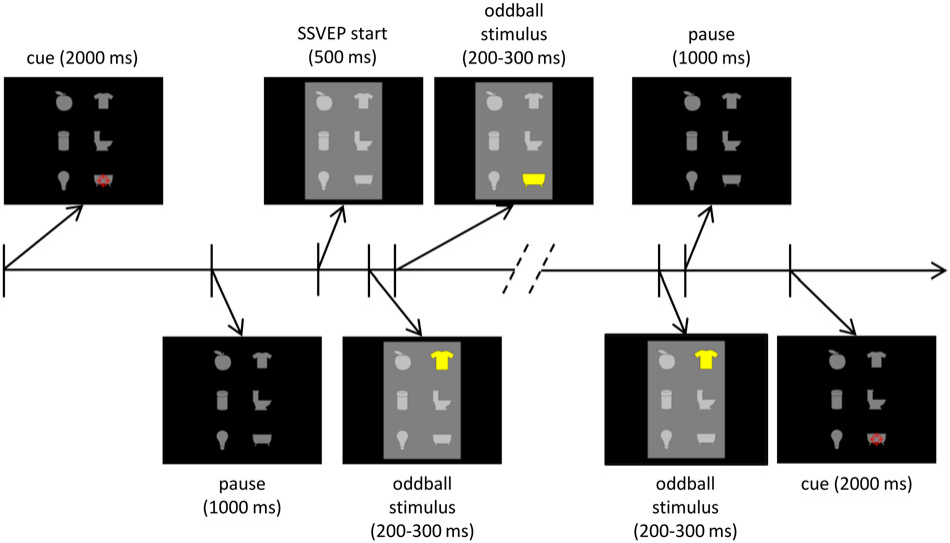
Stimulation cycle for the hybrid condition for the first and second experiment. The stimulation cycle starts with a cue presentation (for 2s), indicating to the participant the target item, followed by a 1s pause during which the cue disappeared and all icons remained gray. A background rectangle then starts to flicker and the oddball stimulation begins 500ms later. The oddball stimulation consists of 10 *flashing sequences* during which each of the 6 icons is flashed one after another in random order for a duration randomly set between 200 and 300ms.

As shown in Fig. 1, a typical *stimulation cycle* (or *trial*), started with a cue presentation (for 2s), indicating to the participant his/her target icon, followed by a 1s pause during which the cue disappeared and all icons remained gray. A background rectangle then started to flicker and the oddball stimulation began 500ms later. The oddball stimulation consisted of 10 *flashing sequences* during which each of the 6 icons was flashed one after another in random order for a duration randomly set between 200 and 300ms (so as to avoid steady-state EEG responses to the oddball stimulation). As is usually done for oddball experiments [1], the participants were instructed to focus on their target icon and count the number of times it is flashed. A 1s pause followed the oddball stimulation and preceded the next cue. An *experimental run* lasted approximately 4 minutes and consisted of 12 consecutive trials, so that each of the 6 icons was cued twice (in random order).

As we aimed here at studying the effect of the flickering background on the P300 response, we considered 5 experimental conditions. One of them (*pure oddball condition*) consisted of a run as described in the previous paragraph but in which no flickering background was displayed. The 4 other conditions (*hybrid conditions*) differed only by the frequency of the flickering background; the frequencies used were 8.57, 10, 12 and 15Hz, corresponding to the refresh rate of the screen (60Hz) divided by 7, 6, 5 and 4, respectively.

For each of the 5 conditions, all subjects performed 3 runs, therefore the whole experiment consisted of 15 runs of approximately 4 minutes each (12 trials per run). The run order was randomized for each subject and a 5 to 10 minutes pause was scheduled every 5 runs.

#### Experiment 2: Pure *vs*. hybrid SSVEP responses

The aim of the second experiment was to study the effect of an oddball stimulation paradigm on the SSVEP responses. Eight subjects participated in the experiment (S10–S17).

The experimental run was the same as for the first experiment. Two experimental parameters were manipulated, the first one was the stimulation frequency; the same frequencies as for the first experiment were used (8.57, 10, 12 and 15Hz). The second experimental parameter was the presence or not of the oddball stimulation sequence. When the oddball stimulation was presented (*hybrid condition*), the participants were instructed to count the number of flashes of the target icon. When no oddball stimulation was displayed (*pure SSVEP condition*), their task was simply to focus on their target icon.

The experiment consisted thus of 8 runs of approximately 4 minutes each (12 trials per run). Each run was characterized by a stimulation frequency and a stimulation type (pure SSVEP or hybrid) so that all combinations of those two factors were covered by the 8 runs. The order of the runs was randomized for each subject and a 5 to 10 minutes pause was set up after the first 4 runs.

#### Experiment 3: hybrid system

The third experiment consisted in a proof-of-concept for a hybrid P300-SSVEP BCI. Eight subjects took part in the experiment (S18–S25).

**Fig 2:**
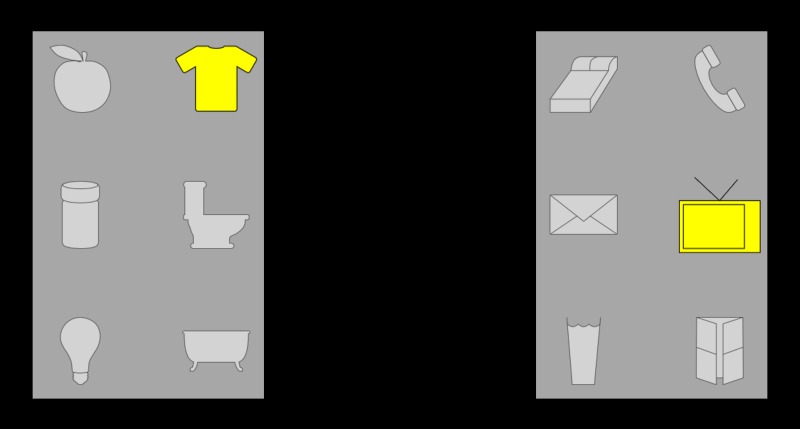
Example of stimulus for the third experiment. Icons from left and right parts of the screen are simultaneously flashed one at a time in random order while the background rectangles are flickering at 12Hz (left) and 15Hz (right).

Two rectangles flickering at 12Hz and 15Hz where simultaneously presented on the left and the right side of the screen, respectively. Within each of those rectangles 6 items were presented so that 2 independent and simultaneous oddball stimulation sequences could be presented on the screen (see Fig. 2). The *stimulation cycle* was the same as described earlier with icons from the left and right rectangles flashing simultaneously, however, their order was set independently (and randomly) for each rectangle. An *experimental run* lasted for approximately 4 minutes and consisted of 12 consecutive trials (with 10 repetitions of the flashing sequence for each), so that each of the 12 icons was cued once (in random order). Each subject participated in 8 consecutive runs with a 5 to 10 minutes pause after the the 4^th^ run.

### Data Analysis

#### Experiment 1: Pure oddball *vs*. hybrid ERPs comparison

We first observed average responses to target stimuli for each of the 5 experimental conditions. The EEG signals were filtered between 0.5 and 20Hz (zero-phase 3^rd^ order Butterworth filter) and epochs were cut from 200ms before the stimuli onsets until 800ms after. In order to ensure that none of the epochs used for averaging were corrupted by ocular artifacts, we rejected, for each *experimental run*, the 10% epochs with the highest peak-to-peak amplitude [10]. We also visually inspected the filtered EEG traces to verify that no ocular artifact could be seen within the 90% remaining epochs. For each participant, averaged ERPs were observed and compared with respect to the experimental condition. We particularly looked for differences between the pure oddball condition and the hybrid conditions (4 other conditions with flickering square).

To assess differences and similarities, we calculated for each subject and EEG channel the correlation coefficient between average ERPs for all *conditions pairs* (5 experimental condition, 10 condition pairs, *e.g*. oddball/hybrid-10Hz, hybrid-12Hz/hybrid-10Hz, *etc*). Those correlation data were modelled with a linear-mixed effect model with the channels nested within subjects as a random effect and the condition pair as a fixed effect. Post-hoc pairwise comparisons for each level of the *conditions pair* factor were conducted via Tukey’s test (using the *multcomp* R package [25]). The significance level was *α* = 0.01.

#### Experiment 1: Pure oddball *vs*. hybrid ERPs classification

The second step was to compare the P300 classification accuracies. The EEG signals were filtered between 0.5 and 20Hz (zero-phase 3^rd^ order Butterworth filter), epochs were cut from the stimuli onsets until 600ms after and downsampled to 128Hz. The resulting epochs were labelled as either *target epochs* or *non-target epochs* according to whether they corresponded to the EEG response to a target stimulus (flashing of a target icon) or a non-target one (flashing of any non-target icon). For each subject and experimental condition, we ran a 3-fold cross-validation where a linear support vector machine (SVM) was trained [26] on the data collected during 2 out of the 3 experimental runs and the performance was measured on every trial of the remaining run. The SVMs themselves were trained via a 10-fold cross-validation and a line search for optimizing the regularization parameter.

For each trial, the SVM returns a score associated with each of the 6 icons and the icon with the highest score identifies the *detected target*. We define a *correctness value* which is set to 1 if the detected target matches the cued icon (correct detection) and 0 otherwise. We thus obtain for each subject and experimental condition 36 correctness values. The correctness values were computed for the number of repetitions *N*_*r*_ of the flashing sequence (varying from 1 to 10). In order to mimic the behavior of a BCI, for each trial and each icon, epochs were averaged over the **N*_*r*_ first repetitions*.

The correctness data were modelled using a logistic mixed effect model [27] with the number of repetitions nested within subjects as random factors. As fixed effects, we considered the experimental condition (5 levels factor), the number of repetitions (10 levels factor) and the interaction between those 2 factors. The p-value for the experimental condition fixed effect was obtained by a *likelihood ratio test* of the full model with the effect in question against the model without the effect in question (and all interactions involving this effect) [23].

#### Experiment 2: SSVEP response analysis

For each trial we estimated the power of the EEG signal measured by the Oz channel in the first, second and third harmonic of the stimulation frequency of the trial.

$$\begin{array}{c} P\left(Y,f\right)={\left(\frac{1}{n_{s}}\sum_{i=1}^{n_{s}}y_{i}\cos\left(2\pi if/f_{s}\right)\right)}^{2}+{\left(\frac{1}{n_{s}}\sum_{i=1}^{n_{s}}y_{i}\sin\left(2\pi if/f_{s}\right)\right)}^{2} \end{array}$$ (1)

where *f* is the frequency of interest in Hz, *f*_*s*_ the sampling frequency of the EEG signal (1024Hz in our case) and *Y* = [*y*_1_, *y*_2_, …, *y*_*n*_*s*__] the vector representing the EEG data.

We obtained thus for each subject, stimulation frequency and stimulation type, 12 power values (number of trials per run) for all three harmonics. The data for each harmonic were analysed with a linear mixed effect regression. The fixed factors considered were the stimulation frequency (4 levels, ordered by increasing frequency, orthogonal polynomial contrasts), the stimulation type (pure oddball *vs*. hybrid) and the interaction between those 2 factors. We considered random intercept for the subject, and for the subject-stimulation frequency and subject-stimulation type interactions. The p-values for the fixed effects were obtained by likelihood ratio tests of the full model with the effect in question against the model without the effect in question. For factors with significant effects, the p-values for the contrasts were obtained via Markov-chain Monte Carlo (MCMC) sampling [28].

#### Experiment 3: classifying hybrid data

For the P300 classification, we used the first 2 runs for training the classifiers and the 6 remaining runs for measuring the detection accuracy. We used the same procedure as described earlier to build a linear SVM classifier on the training data and measure the performance on the test data with respect to the number of repetitions of the flashing sequence. We thus obtain, for each number of repetitions, 72 binary values representing the correctness of detection for each of the 12 trials that compose all 6 testing runs.

For SSVEP detection, the EEG signals were first high-pass filtered above 0.2Hz (zero-phase 4^th^ order Butterworth filter), and downsampled to 256Hz. For the detection itself we used a technique proposed by [29] (also applied by [30, 31]). This technique consists in first applying a spatial filter to the EEG data using the *Minimum Energy Combination* method. It results in a set of linear combinations of the original EEG signals for which the noise is minimized at the frequencies of interest (*i.e*. the 4 stimulation frequencies) and their harmonics (we considered the first three harmonics). In the second step, a scoring function is calculated for each of the 2 stimulation frequencies and the one with the highest score is identified as the *winner frequency*. A correct detection corresponds to the case where the winner frequency matches the actual target frequency. The scoring function corresponds to the average of the signal-to-noise ratios across harmonics and components of the spatially filtered signals. The signal-to-noise ratio is calculated as the ratio of the estimated signal power and the estimated noise power at the desired frequency (see [29] for details).

The icon detection correctness was determined by combining the correctness of the P300 detection and the SSVEP detection as both need to be correctly detected for the target icon to be correctly identified. As the course of the stimulation is constrained by the oddball stimulation scheme, the correctness for P300, SSVEP and icon detection were measured for the *N*_*r*_ first repetitions of the flashing sequence, with *N*_*r*_ varying from 1 to 10.

Besides icon detection accuracy, we also measured the Information Transfer Rate (ITR, see for example [32–35]) expressed in *bits per minute* and defined as:

$$\begin{array}{c} I=B\times \frac{N_{c}}{\sum_{i=1}^{N_{c}}t_{i}}\times 60 \end{array}$$ (2)

where *N*_*c*_ is the number of symbols communicated, *t*_*i*_ the time in seconds needed to communicate the *i* ^*th*^ symbol and *B* the bitrate expressed in bits per symbols and defined by:

$$\begin{array}{c} B=\log_{2}\left(N\right)+p\log_{2}\left(p\right)+\left(1-p\right)\log_{2}\left(\frac{1-p}{N-1}\right) \end{array}$$ (3)

where *p* is the classification accuracy and *N* the number of possible symbols to communicate. For the SSVEP classification problem, *p* is estimated by the division of the number of times the correct SSVEP target was identified by the total number of trials, and *N* is the number of SSVEP targets (*N* = 2). For the P300 classification problem, *p* is estimated by the division of the number of times the correct P300 target was identified by the total number of trials, regardless of the results of the SSVEP classification (if we were to take the SSVEP classification into account, then it would be similar to computing the ITR related to the symbol detection in the hybrid condition). This means that the P300 classifier is always applied to data corresponding to the actual target screen portion and therefore, *N* is the number of possible commands in each screen portion (*N* = 6). Finally, for the hybrid condition, *p* represents the division of the number of times the correct symbol was identified (when both P300 and SSVEP classifiers identify the correct target) by the total number of trials, and *N* the total number of symbols the user can choose from (*N* = 12). As ITR typically reflects online performance and this study relies on offline analyses of EEG, the ITR values reported should be regarded as estimates of expected online performance.

## Results

### Experiment 1: pure oddball *vs*. hybrid ERPs

**Fig 3:**
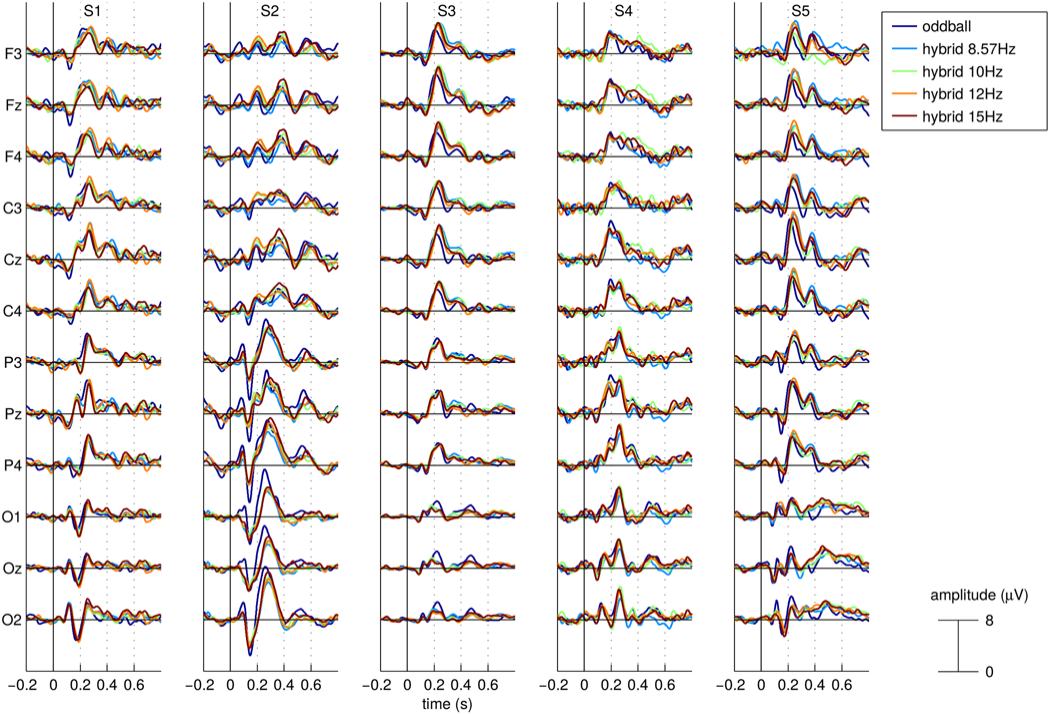
Average ERP responses to the target stimuli. ERPs are shown for a selection of EEG channels covering the scalp from frontal to occipital locations for all 5 experimental conditions and for subjects S01 to S05. Time 0 represents stimuli onset.

**Fig 4:**
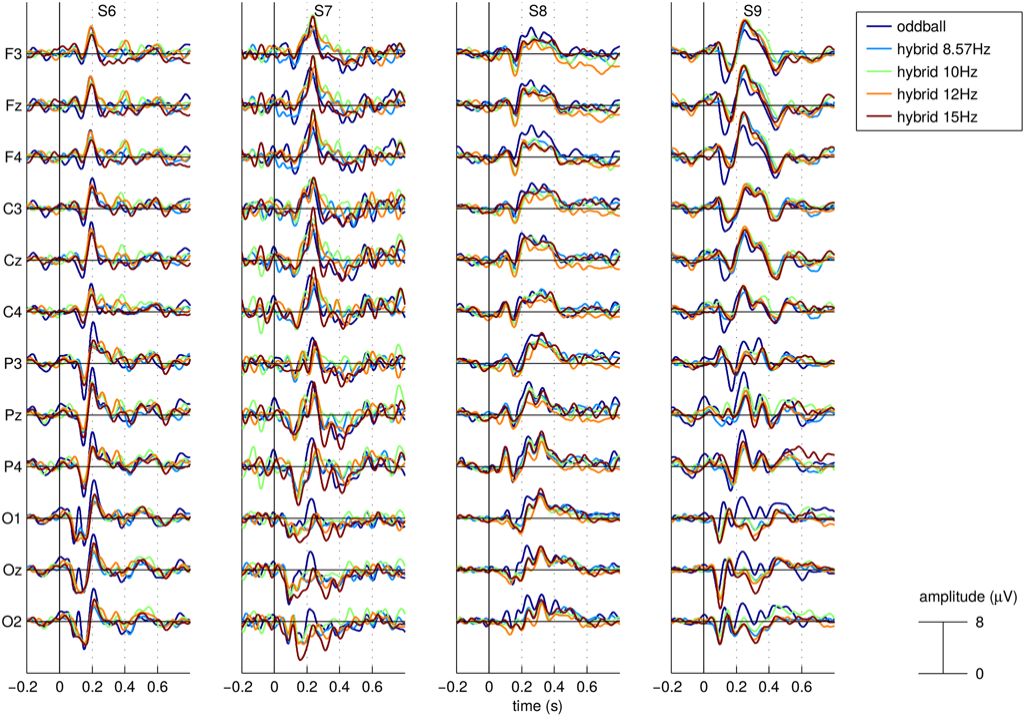
Average ERP responses to the target stimuli. ERPs are shown for a selection of EEG channels covering the scalp from frontal to occipital locations for all 5 experimental conditions and for subjects S06 to S09. Time 0 represents stimuli onset.

The average responses to target stimuli for each of the 5 experimental conditions, all subjects and a selection of EEG channels covering the scalp from frontal to occipital locations are shown in Fig. 3 (subjects S1 to S5) and Fig. 4 (subjects S6 to S9). As observed in other studies (*e.g*.[36, 37]), the target ERP is not only characterized by the P300 component but also by a parieto-occipital negative deflection between 100 and 200ms after the stimulus onset for some subjects. One can also notice that for most subjects and EEG channels, there seem to be systematic differences between, on one side, the targets ERPs of the 4 hybrid conditions and, on the other side, the target ERPs for the pure oddball condition. However, the nature of those differences is not systematic across subjects in terms of scalp location, polarity, latency and amplitude.

For example, it can take the form of a time shift as observed for subjects S3 and S5 in the frontal and central channels, where the positive peak observed between 200 and 300ms appears slightly earlier for the pure oddball condition than for the hybrid conditions. It can also take the form of a difference in peak amplitude as one can see in the ERPs of subject S2 where the negative peak observed in the parietal electrodes between 100 and 200ms is characterized by a larger amplitude in the pure oddball condition than in the hybrid conditions. Similarly, the ERPs of subject S6 in the central and parietal channels are characterized by one negative peak at around 150ms and a positive one at around 200ms, both peaks showing a stronger magnitude in the pure oddball condition than in the hybrid ones.

One can also observe differences in both amplitude and latency, as for the ERPs recorded for subject S2 in the occipital channels where the positive peak between 200 and 300ms appears earlier with a stronger magnitude in the pure oddball condition than in the hybrid ones. Similarly the ERPs recorded for subject S9 in the frontal and central channels between 100 and 200ms appear earlier and have a stronger magnitude for the pure oddball condition than for the hybrid ones.

**Fig 5:**
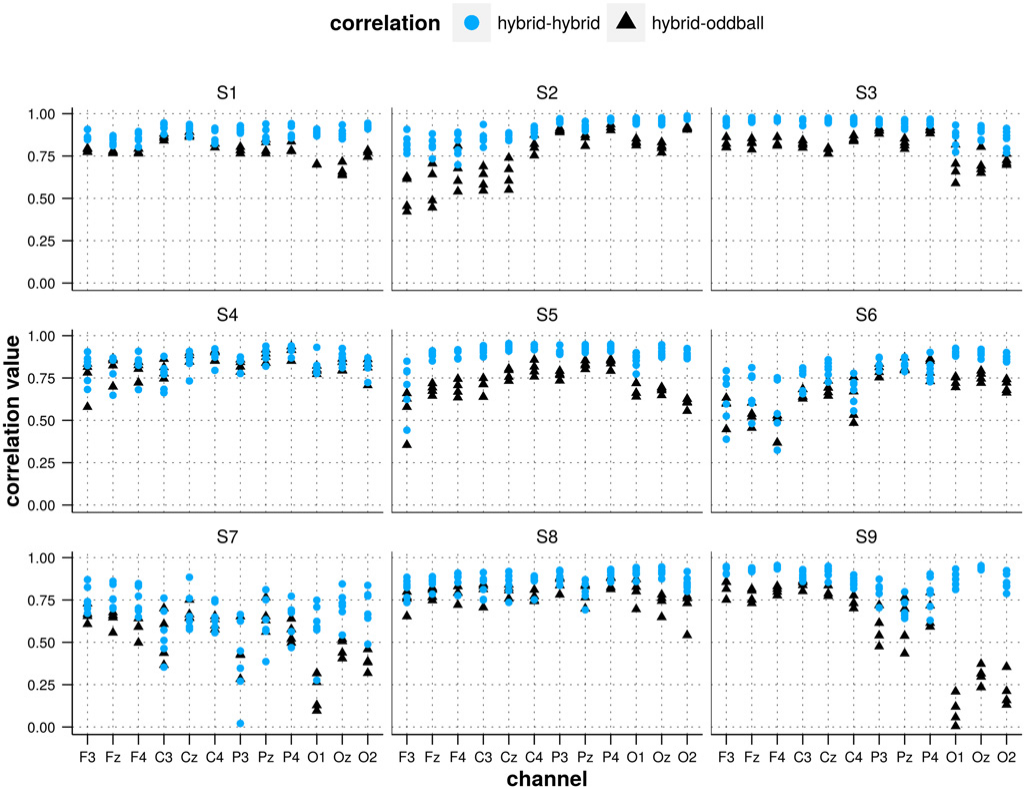
Pairwise correlations between ERPs for all conditions per subject and EEG channel. The triangles (blue) represent correlation values measured between the average ERP recorded in the oddball condition and the average ERP recorded in each of the 4 hybrid conditions while the dots (red) represent correlation values measured between 2 average ERPs recorded in different hybrid conditions.

In Fig. 5, we show for each subject and channel the correlations between the mean ERPs corresponding to each pair of conditions. We distinguish correlations between 2 hybrid ERPs from correlations between the oddball ERP and a hybrid ERP. The figure seems to suggest that the correlation is typically lower when measured between the oddball ERP and an hybrid ERP.

In Table 1, we show the results for the post-hoc pairwise comparisons (Tukey’s test) for the model build on those correlation data (see the methods section for description). It appears that, on the one hand, the correlation between the oddball and every hybrid ERP was always significantly lower than the correlation between any 2 hybrid ERPs while, on the other hand, no significant difference was observed for the correlation between pairs of hybrid ERPs.

**Table 1:** Results from the post hoc pairwise comparisons of the linear mixed model built on the correlation data shown in Fig. 5. The first column represents the tested hypothesis; “odd” represents the oddball condition while “h08”, “h10”, “h12” and “h15” represent the hybrid condition at 8.57, 10, 12 and 15Hz, respectively. Therefore “corr(odd, h10)” represents the correlation between oddball and 10Hz-hybrid ERPs. The first line of the table tests for significance in the difference between, on the one hand, correlation values between ERPs recorded in the oddball and in the 10Hz-hybrid conditions and, on the other hand, correlation values between ERPs recorded in the oddball and in the 8.57Hz-hybrid conditions. The second and third columns represent respectively the test statistic and the associated p-value. the symbol ** denotes statistical significance below 0.01.

Null hypothesis	z-value	p-value	
corr(odd,h10)—corr(odd,h08) == 0	−3.152	0.05146	
corr(odd,h12)—corr(odd,h08) == 0	0.787	0.99877	
corr(odd,h15)—corr(odd,h08) == 0	3.272	0.03570	
corr(h08,h10)—corr(odd,h08) == 0	16.800	< 0.001	**
corr(h08,h12)—corr(odd,h08) == 0	17.596	< 0.001	**
corr(h08,h15)—corr(odd,h08) == 0	15.247	< 0.001	**
corr(h10,h12)—corr(odd,h08) == 0	17.428	< 0.001	**
corr(h10,h15)—corr(odd,h08) == 0	14.885	< 0.001	**
corr(h12,h15)—corr(odd,h08) == 0	17.384	< 0.001	**
corr(odd,h12)—corr(odd,h10) == 0	3.939	0.00337	**
corr(odd,h15)—corr(odd,h10) == 0	6.423	< 0.001	**
corr(h08,h10)—corr(odd,h10) == 0	19.952	< 0.001	**
corr(h08,h12)—corr(odd,h10) == 0	20.748	< 0.001	**
corr(h08,h15)—corr(odd,h10) == 0	18.399	< 0.001	**
corr(h10,h12)—corr(odd,h10) == 0	20.580	< 0.001	**
corr(h10,h15)—corr(odd,h10) == 0	18.037	< 0.001	**
corr(h12,h15)—corr(odd,h10) == 0	20.536	< 0.001	**
corr(odd,h15)—corr(odd,h12) == 0	2.485	0.27636	
corr(h08,h10)—corr(odd,h12) == 0	16.013	< 0.001	**
corr(h08,h12)—corr(odd,h12) == 0	16.809	< 0.001	**
corr(h08,h15)—corr(odd,h12) == 0	14.460	< 0.001	**
corr(h10,h12)—corr(odd,h12) == 0	16.641	< 0.001	**
corr(h10,h15)—corr(odd,h12) == 0	14.098	< 0.001	**
corr(h12,h15)—corr(odd,h12) == 0	16.597	< 0.001	**
corr(h08,h10)—corr(odd,h15) == 0	13.528	< 0.001	**
corr(h08,h12)—corr(odd,h15) == 0	14.324	< 0.001	**
corr(h08,h15)—corr(odd,h15) == 0	11.975	< 0.001	**
corr(h10,h12)—corr(odd,h15) == 0	14.156	< 0.001	**
corr(h10,h15)—corr(odd,h15) == 0	11.614	< 0.001	**
corr(h12,h15)—corr(odd,h15) == 0	14.112	< 0.001	**
corr(h08,h12)—corr(h08,h10) == 0	0.796	0.99865	
corr(h08,h15)—corr(h08,h10) == 0	−1.553	0.87041	
corr(h10,h12)—corr(h08,h10) == 0	0.628	0.99981	
corr(h10,h15)—corr(h08,h10) == 0	−1.915	0.65872	
corr(h12,h15)—corr(h08,h10) == 0	0.584	0.99989	
corr(h08,h15)—corr(h08,h12) == 0	−2.349	0.35791	
corr(h10,h12)—corr(h08,h12) == 0	−0.168	1.00000	
corr(h10,h15)—corr(h08,h12) == 0	−2.711	0.16840	
corr(h12,h15)—corr(h08,h12) == 0	−0.212	1.00000	
corr(h10,h12)—corr(h08,h15) == 0	2.181	0.47009	
corr(h10,h15)—corr(h08,h15) == 0	−0.362	1.00000	
corr(h12,h15)—corr(h08,h15) == 0	2.137	0.50033	
corr(h10,h15)—corr(h10,h12) == 0	−2.543	0.24583	
corr(h12,h15)—corr(h10,h12) == 0	−0.044	1.00000	
corr(h12,h15)—corr(h10,h15) == 0	2.499	0.26900	

### Experiment 1: classifying ERPs

The results from the previous section support the observations made from Fig. 3 that although the differences between ERPs recorded in different hybrid conditions do not seem to differ much, there appears to be clear differences between the oddball ERPs and the hybrid ones. However, from a BCI point of view, the question is not so much about the differences in the shape of the ERPs but more about differences in classification accuracy. We aim here at assessing whether the classification accuracy varies significantly from one condition to the other.

**Fig 6:**
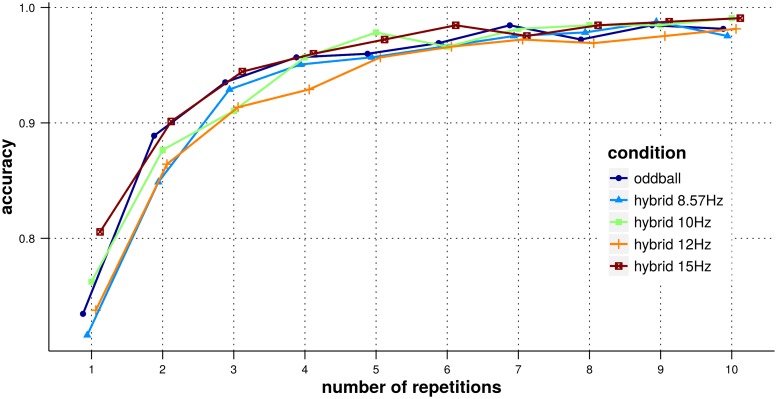
Accuracy of P300 ERP detection. The x-axis represents the number of repetitions, results are shown for each experimental condition and detection accuracies are averaged over subjects.

The detection accuracy (averaged across subject) with respect to the number of repetitions considered is shown in Fig. 6. As is typical for P300-based BCIs, we observe an increase in detection accuracy with respect to the number of repetitions. The figure does not suggest any clear and systematic difference in accuracy between the 5 experimental conditions. This observation was supported by our statistical analysis (see description in the methods section), no significant effect of the experiment condition on the detection correctness was observed (*χ* ^2^(40) = 40.1, *p* = 0.47).

Therefore, and despite the differences between oddball and hybrid ERPs reported in the previous section, the oddball detection does not appear to be affected by neither the presence of the SSVEP stimulation, nor the frequency of the SSVEP stimulation (at least for the 4 frequencies tested).

### Experiment 2: pure *vs*. hybrid SSVEPs responses

**Fig 7:**
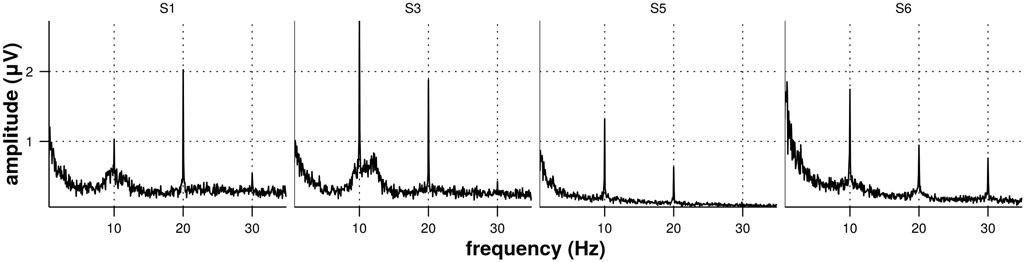
Amplitude spectrum of SSVEP responses. The figures represent the amplitude spectrum (average over 12 trials) of the EEG signals recorded at Oz for subjects S1, S3, S5 and S6 with a 10Hz stimulation frequency for the pure SSVEP condition.

In Fig. 7, we show the mean amplitude spectrum (calculated via Fast Fourier Transform—FFT) recorded at channel Oz across trials for 4 participants and the 10Hz stimulation frequency in the pure SSVEP condition. One can observe distinctive peaks in the FFT spectrum corresponding to the stimulation frequency and its second and third harmonics. The figure shows that the first harmonic is not necessarily the one with the strongest response (subject S1) and that not all subjects will produce a response in all three harmonics.

**Fig 8:**
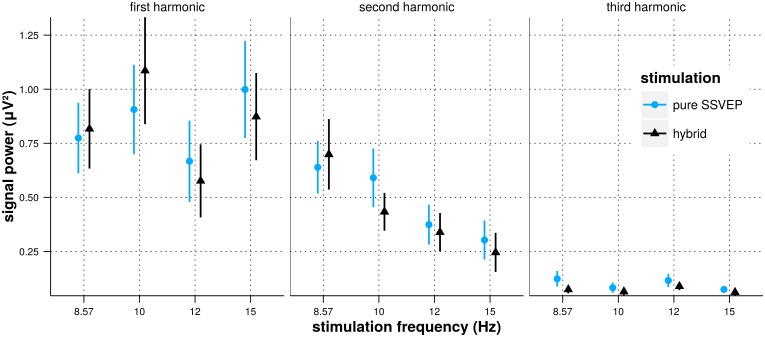
Power of the EEG signal recorded at Oz during the SSVEP stimulation with respect to stimulation frequency and stimulation type. The values are averaged over trials and subjects in every conditions and the bars represent the 95% confidence intervals.

In Fig. 8 we show the power of the EEG signals measured at the scalp position Oz in the first, second and third harmonic of the stimulation frequency (subject and trial means) with respect to the stimulation frequency and the stimulation type.

For the first harmonic, no significant effect on the signal power was found for neither the stimulation type (pure SSVEP *vs*. hybrid, *χ* ^2^(1) = 0.0004, *p* = 0.98), nor the stimulation frequency (*χ* ^2^(3) = 2.54, *p* = 0.47), nor the interaction between them (*χ* ^2^(3) = 4.4, *p* = 0.22).

Concerning the second harmonic, no significant effect on the signal power was found for neither the stimulation type (pure SSVEP *vs*. hybrid, *χ* ^2^(1) = 0.552, *p* = 0.46), nor the interaction between stimulation type and stimulation frequency (*χ* ^2^(3) = 5.59, *p* = 0.13). However, we observed a significant effect of the stimulation frequency on the signal power (*χ* ^2^(3) = 10.6, *p* = 0.014). The contrast analysis (MCMC sampling) revealed a significant linear trend between the stimulation frequency and the signal power (*p*_*mcmc*_ < 0.001) and no significant quadratic or cubic trend (*p*_*mcmc*_ = 0.64 and *p*_*mcmc*_ = 0.85, respectively). As the stimulation frequency was treated as a 4 levels factor (and not a continuous variable) by the statistical model, we do not conclude about the existence of a possible linear relationship between the power of the EEG signal and the stimulation frequency as continuous variables. However, the model does identify a monotonic decrease of the power of the EEG response in the second harmonic with increasing frequency.

For the third harmonic, as for the first harmonic, no significant effect on the signal power was found for the stimulation type (pure SSVEP *vs*. hybrid, *χ* ^2^(1) = 3.37, *p* = 0.07), the stimulation frequency (*χ* ^2^(3) = 4.09, *p* = 0.25), or for the interaction between them (*χ* ^2^(3) = 4.5, *p* = 0.22).

These results show that the power of the EEG measured at Oz in the 3 first harmonics of the SSVEP stimulation frequency is not influenced by the presence of the oddball stimulation. While the stimulation frequency did not appear to influence the power in the first and third harmonics of the SSVEP stimulation frequency, we observed a significant monotonic decrease of the power in the second harmonic as the frequency increases (at least for the 4 frequencies tested).

### Experiment 3: classifying hybrid data

**Fig 9:**
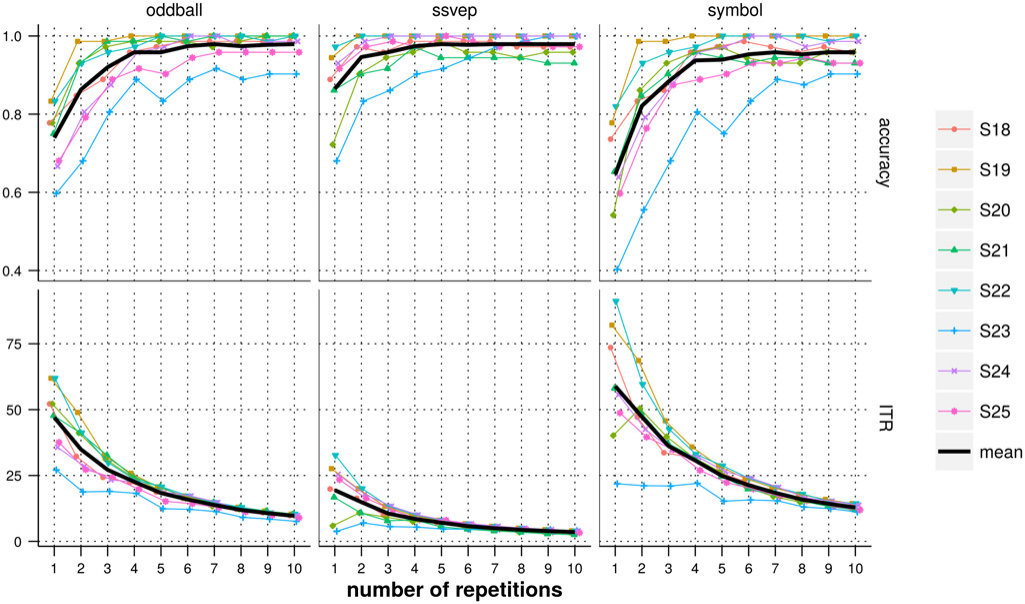
Detection accuracies (top) and ITR values (bottom) with respect to the number of repetitions considered for each subject for the ERP (left), SSVEP frequency (middle) and target icon (left). The black lines represent the average over all subjects. One repetition of the stimulation cycle lasted for 1.5s, the stimulation duration for a number *n*_*r*_ of repetitions is thus 1.5×*n*_*r*_ seconds.

In Fig. 9, we show the detection accuracies and estimated ITR values, for oddball ERP, SSVEP and target icon as a function of the number of repetitions considered, for all subjects individually and averaged across subjects. For a target icon to be correctly detected, both the oddball ERP and the SSVEP target frequency need to be correctly identified, hence, the target icon detection accuracy is bounded by both oddball ERP and SSVEP detection accuracies. Results show that 2 repetitions of the flashing sequence (3s of stimulation) were enough for all subjects except S06 to reach a target icon detection accuracy above 70%, which is commonly accepted as a minimum criterion level necessary for communication [6, 15, 38, 39].

Although the target icon detection accuracy is necessarily lower than the ones of oddball ERP and SSVEP detection, it offers a larger choice of items to communicate than the two modalities individually (12 *versus* 6 for the oddball ERP detection and 2 for the SSVEP detection). As shown in Fig. 9, the ITR values were typically higher for target icon detection than for oddball ERP and SSVEP detection.

## Discussion and conclusion

### Optimizing the SSVEP classification

As shown by the results of the second experiment (see the results section and Fig. 7), the number of harmonics showing a SSVEP response and the harmonic in which the response is the strongest varies across subjects. The SSVEP classification method applied to the data from the third experiment relies on SNR values averaged across harmonics. Therefore, with this method, for each subject, the SNR from each harmonic is of equal importance for determining the target frequency.

This SSVEP classifier does not require any training. However, the oddball classifier does require training data and therefore, training data are also available for SSVEP classification. In this section, we study the possibility to improve the SSVEP classification accuracy by using training data to find optimal weights of the SNRs for each harmonic, specifically for each subject.

We used data from the third experiment. The 2 first runs were used for training and the detection accuracy was measured on the 6 remaining runs (test data). Unlike for the original method, we did not average the SNRs across harmonics, instead, we kept the SNRs from all three harmonics (three dimensional feature space) and used the data to train a linear SVM for each subject (leave-one-out cross-validation and a line search for optimizing the regularization parameter). The linear SVM was then applied on the test data and, for each trial, the frequency for which the SVM output was the highest was identified as the *winner frequency*.

**Fig 10:**
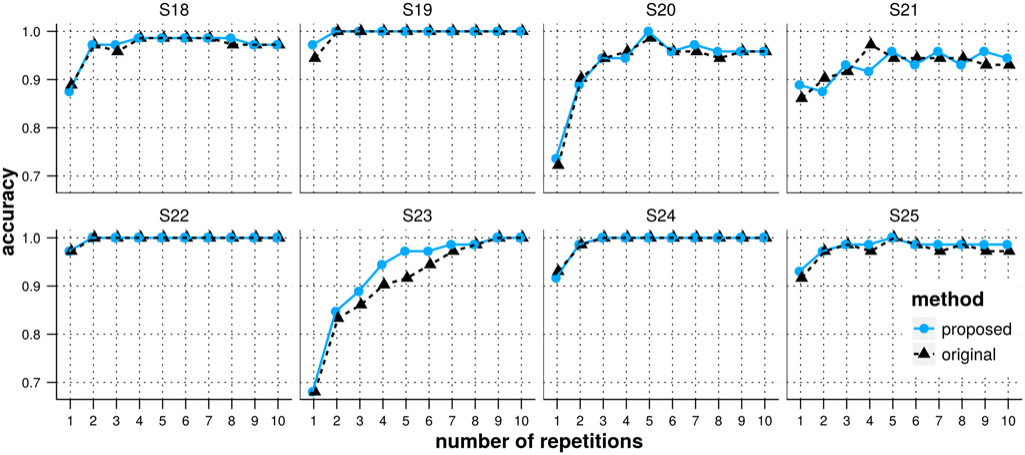
Accuracy of SSVEP detection. The x-axis represents the number of repetitions. Results are shown for all subjects and for both the original method averaging SNRs across harmonics (red) and the proposed method training an SVM for each subject in order to weight optimally each harmonic (blue).

In Fig. 10 we show for each subject the detection accuracy with respect to the number of repetitions of the oddball flashing sequence considered for both the original and the modified method. As with the original method, subjects S18, S19, S22, S24 and S25 reach an accuracy above 95% after 2 repetitions of the stimulation sequence, there is little room for improvement with the proposed method. We observe nevertheless that it performs at least as good as the original one. Concerning the other subjects (S20, S21 and S23), both methods seem to perform equally well with a slight improvement for the proposed method for subject S23 and a number of repetitions of the stimulation sequence between 3 and 6.

Thus, while using training data to obtain subject-specific optimized weights for each harmonic results in an SSVEP detection accuracy that is at least as good as with the original method without training, the improvements realized on the tested population are rather limited.

### Conclusion

The results from our first two experiments suggest that a hybrid visual P300-SSVEP BCI with both stimulus paradigm physically overlapping would not compromise the detection of any of the 2 types of evoked responses.

Our third experiment confirmed those results showing the possibility of detecting both P300 component and SSVEP activity simultaneously, and the results obtained suggest not only the feasibility of such a hybrid BCI system but also a possible advantage over pure P300- and SSVEP-based ones in terms of communication rate.

All the results obtained in our study were obtained from offline analyses of the EEG data. Therefore, further work should report on the feasibility of such hybrid P300-SSVEP BCI in an online setting and focus on comparing the performance obtained with respect to the online performance of equivalent systems purely based on P300 and SSVEP. As our proof-of-concept experiment was performed with only 2 SSVEP stimuli, future online studies should also investigate designs with larger number of SSVEP stimuli and report on the influence of the amount of SSVEP stimuli on both performance and user comfort. If successful, such systems are expected to increase the number of choices an P300-based BCI offers without decreasing the communication speed as well as to overcome the limitation in the number of stimuli that can be displayed on-screen for SSVEP-based BCIs.
